# Efficacy of Dexmedetomidine Added to Ropivacaine for Four-Quadrant Transversus Abdominis Plane Blocks on Hemodynamic Response and Postoperative Analgesia in Laparoscopic Cholecystectomy

**DOI:** 10.7759/cureus.110335

**Published:** 2026-06-06

**Authors:** Manjunath S Kore, Priyanka Shrivastava, Mukesh Kumar, Pratibha A Xess, Alka Lakra

**Affiliations:** 1 Anaesthesiology, Rajendra Institute of Medical Sciences, Ranchi, IND; 2 Anaesthesiology, Samford Hospital, Ranchi, IND

**Keywords:** analgesia, cholecystectomy, dexmedetomidine, hemodynamics, ropivacaine, transversus abdominis plane block

## Abstract

Background

Laparoscopic cholecystectomy, though minimally invasive, is associated with a significant perioperative stress response and postoperative pain. Ultrasound-guided transversus abdominis plane (TAP) block is an effective regional analgesic technique, and the addition of adjuvants such as dexmedetomidine may enhance its efficacy. The aim of the present study is to evaluate the efficacy of adding dexmedetomidine to ropivacaine in four-quadrant ultrasound-guided TAP blocks on intraoperative hemodynamic responses and postoperative analgesia in patients undergoing laparoscopic cholecystectomy.

Materials and methods

This prospective, randomised, double-blind, controlled study was conducted at a tertiary care centre. A total of 126 American Society of Anesthesiologists physical status I and II patients undergoing elective laparoscopic cholecystectomy were randomised into three groups (n=42 each): Group RD received a TAP block with 0.2% ropivacaine plus dexmedetomidine 0.5 µg/kg; Group R received a TAP block with 0.2% ropivacaine alone; and Group C served as a control group and did not receive a TAP block. Intraoperative heart rate and blood pressure were recorded at predefined intervals. Postoperative pain was assessed using the visual analogue scale (VAS), and the time to the first dose of rescue analgesia was recorded.

Results

Demographic parameters were comparable across the groups. Intraoperative heart rate, systolic blood pressure, diastolic blood pressure, and mean arterial pressure were significantly lower in Group RD than in Groups R and C at most time intervals (p < 0.05). Postoperative VAS scores at one and four hours were significantly lower in Group RD (p < 0.001), and the time to the need for rescue analgesia was longer in Group RD than in the other groups.

Conclusion

The addition of dexmedetomidine to ropivacaine in ultrasound-guided TAP blocks provides superior intraoperative hemodynamic stability and enhanced postoperative analgesia compared with ropivacaine alone or no TAP block in patients undergoing laparoscopic cholecystectomy.

## Introduction

Laparoscopic cholecystectomy is the gold standard for managing symptomatic gallbladder disease due to its advantages of minimal invasiveness, reduced postoperative pain, early ambulation, and a shorter hospital stay compared with open cholecystectomy [1]. Despite these benefits, laparoscopic surgery is associated with significant hemodynamic alterations secondary to pneumoperitoneum and patient positioning. Carbon dioxide insufflation increases intra-abdominal pressure and arterial carbon dioxide levels, thereby stimulating sympathetic activity and causing tachycardia, hypertension, and increased systemic vascular resistance [2]. These physiological responses may be poorly tolerated, especially in patients with limited cardiovascular reserve, making attenuation of the stress response an essential component of anaesthetic management [3]. Effective postoperative analgesia is pivotal to enhanced recovery after surgery. Inadequate pain control can activate neuroendocrine stress pathways, delay mobilization, prolong hospital stay, and increase postoperative morbidity [4]. Although opioids remain the mainstay for postoperative analgesia, their use is often limited by adverse effects, such as nausea, vomiting, sedation, respiratory depression, and ileus [5]. Hence, multimodal analgesia strategies incorporating regional anaesthesia techniques have gained popularity to reduce opioid consumption and improve postoperative outcomes [6]. The transversus abdominis plane (TAP) block is a regional anaesthesia technique that provides somatic analgesia to the anterior abdominal wall by blocking intercostal nerves (T7-L1) within the fascial plane between the internal oblique and transversus abdominis muscles [7]. In this study, an ultrasound-guided four-quadrant TAP blocks were administered. First, it was performed bilaterally in the subcostal region, and then bilaterally via the lateral approach to the TAP block. Studies on the four-quadrant TAP block in laparoscopic cholecystectomy are sparse. Dexmedetomidine, when used as an adjuvant in regional anaesthesia, has been shown to prolong analgesia, reduce opioid requirements, and attenuate perioperative hemodynamic responses [8]. Although several studies have evaluated dexmedetomidine as an adjuvant in TAP blocks, data on its efficacy in four-quadrant ultrasound-guided TAP blocks for attenuating intraoperative hemodynamic responses and improving postoperative analgesia in laparoscopic cholecystectomy remain limited. The primary objective of this study was to compare the efficacy of the four-quadrant TAP block using ropivacaine with and without dexmedetomidine in alleviating hemodynamic changes across groups intraoperatively, and the secondary objective was to compare the quality of postoperative analgesia using the visual analogue scale (VAS) and to record the time to the first rescue analgesia.

## Materials and methods

**Figure 1:**
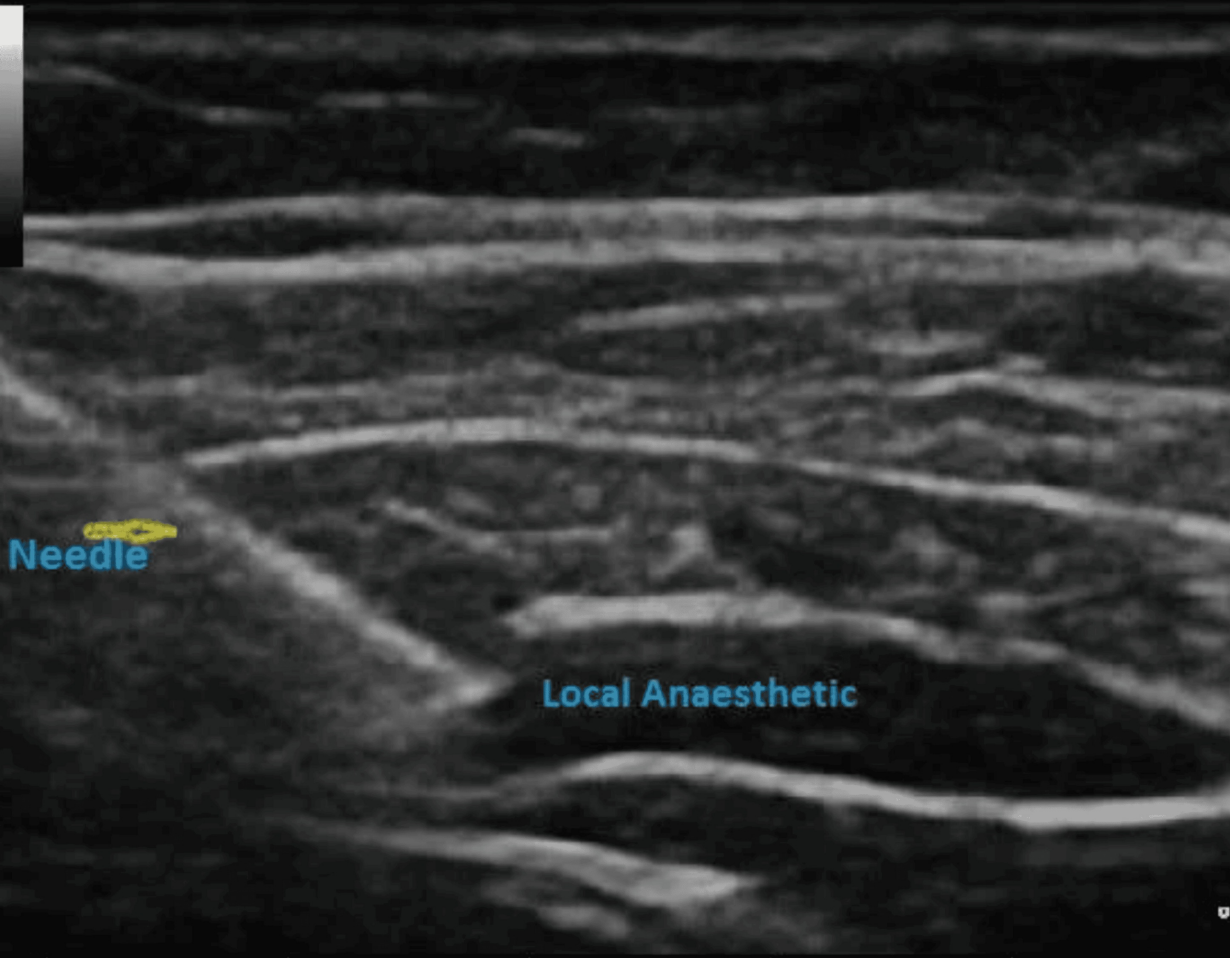
Transversus abdominis plane block

This prospective, randomised, single-blind, controlled trial was conducted at a tertiary care centre over 1.5 years, following Institutional Ethics Committee approval and registration with the Clinical Trials Registry of India (CTRI/2024/01/061166). Written informed consent was obtained from all participants in both Hindi and English prior to enrolment. A total of 126 patients belonging to the American Society of Anesthesiologists (ASA) physical status I and II, scheduled for elective laparoscopic cholecystectomy under general anaesthesia, were enrolled. ASA physical status was defined according to the American Society of Anesthesiologists [9]. Patients were randomly allocated into three groups of 42 patients each. Patients willing to provide written informed consent, aged 18-60 years, either gender, ASA physical status I or II, scheduled for laparoscopic cholecystectomy, body weight >50 kg, and not receiving antiplatelet or anticoagulant therapy were included in the study. Patients were excluded if they refused consent, had a known allergy to any study drug, or had a coagulation disorder (INR > 1.5). Randomisation was performed using a computer-generated random number table. Allocation concealment was ensured by using opaque, sealed envelopes, which were opened in the operating theatre. The study used a single-blind design, in which the patient was unaware of which group he belonged to. Patients were allocated into three groups: Group RD received an ultrasound-guided TAP block with 0.2% ropivacaine (15 mL per quadrant) plus total dexmedetomidine 0.5 µg/kg, equally distributed across all quadrants. Group R received an ultrasound-guided TAP block with 0.2% ropivacaine (15 mL per quadrant), and Group C was the control group receiving no TAP block. In group Rand RD, the total dose of ropivacaine used was 120 mg, and the total dose of dexmedetomidine used in group RD was 0.5 µg/kg. All patients underwent a detailed pre-anaesthesia evaluation one day before surgery, including a history and general and systemic examinations. All routine investigations were done. Patients were kept nil per os for six hours before surgery. Intravenous access was secured with a 20-G cannula. Premedication included intravenous midazolam 0.02 mg/kg and fentanyl 2 µg/kg administered 10 minutes before induction. Standard monitoring (ECG, non-invasive blood pressure, pulse oximetry, and ETCO₂) was instituted. After pre-oxygenation, anaesthesia was induced with propofol 2 mg/kg, and neuromuscular blockade was achieved with vecuronium 0.1 mg/kg. Endotracheal intubation was performed with an appropriately sized cuffed tube. Anaesthesia was maintained with oxygen, nitrous oxide, and sevoflurane, with additional doses of vecuronium as required. After induction of general anaesthesia, four-quadrant ultrasound-guided TAP blocks were performed in patients in Groups RD and R. The subcostal TAP block was performed by placing the ultrasound probe parallel to and inferior to the costal margin and tracing it to the anterior axillary line. A 100-mm Stimuplex needle was inserted from a medial to lateral direction by the in-plane technique to deposit 15 mL of drug between the internal oblique and transversus abdominis muscle. The same was repeated on the other side. Under strict aseptic precautions, a high-frequency linear ultrasound probe (10-5 MHz) was placed transversely at the midaxillary line, between the costal margin and iliac crest. The external oblique, internal oblique, and transversus abdominis muscles were identified. A 100-mm Stimuplex needle was introduced in-plane and advanced under ultrasound guidance into the fascial plane between the internal oblique and transversus abdominals muscles. After negative aspiration, the study drug was injected (15 mL per quadrant), with adequate spread confirmed sonographically on both sides (Figure 1).

Intraoperative hemodynamic parameters, including heart rate, systolic blood pressure, diastolic blood pressure, mean arterial pressure, and ETCO₂, were recorded before starting induction of anaesthesia (baseline) and at five-minute intervals up to the end of surgery. Postoperative pain was measured using the VAS, a validated, freely available tool that does not require a licence for pain assessment [10]. This scale is a 10-cm horizontal line, with 0 indicating no pain and 10 the worst pain imaginable. Before surgery, we explained to patients how to use the scale and asked them to mark the spot that matched their pain level. We then recorded the score in centimetres, assigning the values of 0 to 10 at 1, 4, 8, and 12 hours postoperatively. Rescue analgesia was administered when the VAS exceeded 3. Intravenous paracetamol 1 g was given and repeated every eight hours. If the VAS score remained > 3 despite giving paracetamol, tramadol 1 mg/mL in 100 mL of normal saline was administered. Postoperative nausea and vomiting (PONV) were assessed. Rescue antiemetic therapy with intravenous ondansetron 4 mg was administered as required.

Sample size estimation was based on a previous study by Qin et al., assuming a mean difference of 2.6 and a 2-point difference between the intervention groups [11]. Assuming a type I error (α) of 5% and power (1−β) of 80%, the calculated sample size was 39 patients per group using the Stata power and sample size calculator. Stata is a licensed statistical software (StataCorp LLC, College Station, TX), and access was obtained through an individual license [12]. After accounting for a 10% attrition rate, the final sample size was fixed at 42 patients per group.

Statistical analysis was performed using the Statistical Product and Service Solutions (SPSS, version 22.0; IBM SPSS Statistics for Windows, Armonk, NY) [13]. International Business Machine SPSS Statistics is licensed statistical software (IBM Corp., Armonk, NY), and access was obtained through an individual license. Descriptive statistics were reported as mean and standard deviation for continuous variables and as frequency and percentage for categorical variables. Intergroup comparisons of continuous variables among more than two groups were carried out using one-way analysis of variance (ANOVA). A p-value of less than 0.05 was considered statistically significant. Systolic blood pressure, diastolic blood pressure, mean arterial pressure, heart rate, postoperative pain assessment, and time to rescue analgesia were assessed using one-way ANOVA.

## Results

**Figure 2:**
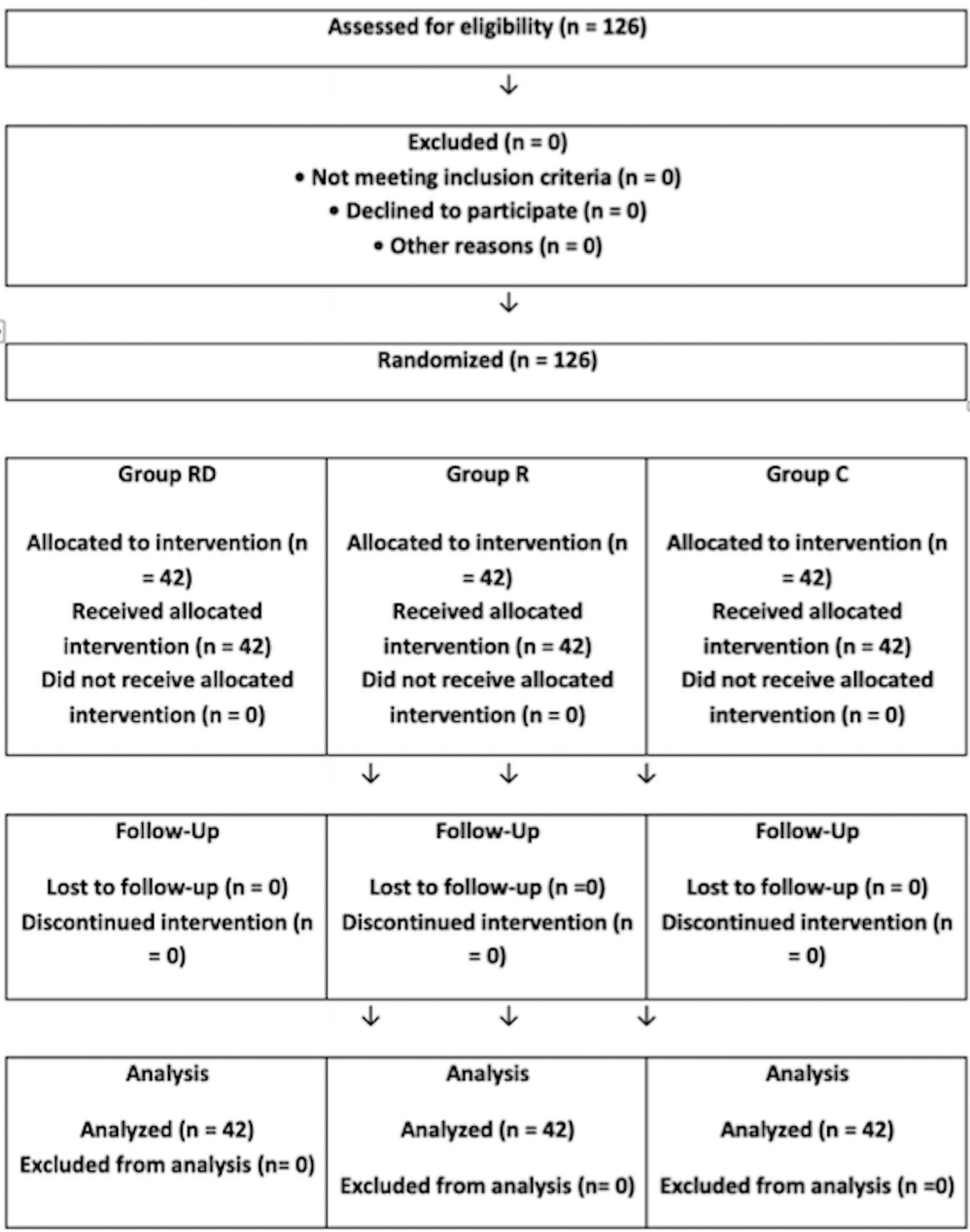
Consolidated Standards of Reporting Trials (CONSORT) flow diagram of participants

A total of 126 patients with ASA physical status I or II, scheduled for laparoscopic cholecystectomy, were enrolled and randomised into three groups (n = 42 each): Group RD (ropivacaine with dexmedetomidine), Group R (ropivacaine alone), and Group C (control). All patients completed the study and were included in the final analysis. The Consolidated Standards of Reporting Trials (CONSORT) flowchart is shown in Figure 2.

Mean Age and Mean Weight Between the Groups

There were no statistically significant differences in the age (p = 0.209) and weight (p = 0.684) distributions among the three groups (Table 1).

**Table 1:** Comparison of the mean age and mean weight across groups

Parameter	Group R (mean ± SD)	Group RD (mean ± SD)	Group C (mean ± SD)	F‑value	p‑value	
Age (years)	41.67 ± 11.879	38.07 ± 10.962	41.95 ± 10.500	1.59	0.209	
Weight (kg)	60.00 ± 7.095	59.12 ± 7.362	60.43 ± 6.534	0.38	0.684	
Test used: One-way ANOVA						

Gender Distribution

There was no statistical difference in the gender distribution (p = 0.139) (Table 2).

**Table 2:** Gender distribution across groups

Sex	Group R (n=42)	Percentage	Group RD (n=42)	Percentage	Group C (n=42)	Percentage	Test Statistic	p‑value
Male	11	26.20%	5	11.90%	12	28.57%	(χ²)=3.97	0.139
Female	31	73.80%	37	88.09%	30	71.42%		
Test used: Chi-square test								

Systolic Blood Pressure and Diastolic Blood Pressure

There was no statistically significant difference in baseline systolic or diastolic blood pressure across all three groups (Table 3). Significant differences in systolic and diastolic blood pressure were observed at 15, 30, 45, 60, and 90 minutes (p < 0.05) (Table 3).

**Table 3:** Intraoperative systolic blood pressure and diastolic blood pressure Test used: One-way ANOVA

Time Interval	Group R (mean ± SD)	Group RD (mean ± SD)	Group C (mean ± SD)	Test Statistic (F)	p‑value
Systolic BP					
Baseline	122.69 ± 9.623	121.62 ± 10.695	121.83 ± 13.492	0.104	0.901
15 min	125.52 ± 12.673	121.12 ± 10.498	118.60 ± 11.372	3.95	0.023
30 min	126.38 ± 9.044	123.31 ± 8.351	120.50 ± 8.716	4.71	0.01
45 min	125.67 ± 7.039	123.98 ± 8.161	120.67 ± 9.036	4.12	0.019
60 min	127.21 ± 8.150	123.26 ± 7.677	121.41 ± 8.637	5.47	0.005
90 min	128.27 ± 6.918	124.26 ± 5.471	123.00 ± 7.303	4.71	0.01
Diastolic BP					
Baseline	80.69 ± 6.773	79.67 ± 9.527	77.12 ± 10.484	1.73	0.182
15 min	81.05 ± 9.255	79.33 ± 9.406	75.10 ± 9.396	4.56	0.013
30 min	80.81 ± 10.196	81.43 ± 7.006	74.55 ± 7.198	7.21	<0.001
45 min	78.93 ± 8.637	81.55 ± 6.348	76.38 ± 6.677	5.29	0.006
60 min	79.19 ± 8.660	79.98 ± 7.511	74.98 ± 7.441	4.71	0.01
90 min	79.37 ± 8.672	79.68 ± 5.492	82.86 ± 5.276	4.95	0.008

Heart Rate and Mean Arterial Pressure

The baseline heart rate was comparable across the three groups (p = 0.763). At 15, 30, 45, 60, and 90 minutes, a statistically significant difference was observed, with higher heart rates in the control group than in Groups R and RD (p < 0.05 at all intervals) (Table 4). Mean arterial pressure was comparable across all groups at baseline (p=0.2). Significant differences were observed in mean arterial pressure at all intraoperative time intervals (p < 0.05) (Table 4). Mean arterial pressure was significantly lower in group R and RD than in group C at 15, 30, 45, and 90 minutes.

**Table 4:** Heart rate and mean arterial pressure (MAP) across groups Test used: One-way ANOVA

Parameter	Time Interval	Group R (Mean ± SD)	Group RD (Mean ± SD)	Group C (Mean ± SD)	Test Statistic (F)	p‑value
Heart Rate	Baseline	83.62 ± 9.451	82.38 ± 7.499	83.83 ± 11.820	0.27	0.763
	15 min	83.19 ± 9.308	83.26 ± 6.085	90.31 ± 10.753	7.21	<0.001
	30 min	82.81 ± 7.890	84.33 ± 7.811	88.69 ± 10.266	4.98	0.007
	45 min	82.98 ± 7.563	84.76 ± 6.585	88.10 ± 8.021	5.01	0.007
	60 min	83.90 ± 6.973	83.12 ± 6.946	88.66 ± 6.394	6.89	0.001
	90 min	84.00 ± 6.119	83.52 ± 4.304	88.23 ± 7.191	4.56	0.011
MAP	Baseline	94.69 ± 5.53	93.65 ± 6.98	92.02 ± 7.78	1.63	0.20
	15 min	84.76 ± 9.406	76.83 ± 7.460	81.05 ± 11.778	7.12	0.001
	30 min	82.26 ± 8.884	77.31 ± 5.073	81.10 ± 10.583	3.91	0.023
	45 min	79.00 ± 10.585	75.14 ± 4.052	80.81 ± 8.821	5.21	0.007
	60 min	82.07 ± 7.869	77.60 ± 6.367	79.93 ± 9.199	3.42	0.037
	90 min	85.03 ± 7.237	79.61 ± 4.209	87.59 ± 7.398	8.34	<0.001

Postoperative Pain Assessment

The VAS scores at one and four hours postoperatively were significantly lower in Group RD than in Groups R and C (p < 0.001) [10]. At eight hours, no statistically significant difference was observed among the groups (p = 0.276) (Table 5).

**Table 5:** Postoperative visual analogue scores Test used: One‑way ANOVA with Bonferroni post‑hoc correction was used for intergroup comparisons

Time Interval	Group R (Mean ± SD)	Group RD (Mean ± SD)	Group C (Mean ± SD)	Test Statistic (F)	p‑value	Bonferroni Post‑hoc Results
1 hour	2.50 ± 1.042	1.98 ± 0.715	4.86 ± 1.775	15.42	< 0.001	R vs RD: Not Significant
						R vs C: p < 0.001
						RD vs C: p < 0.001
4 hours	5.54 ± 0.951	3.54 ± 1.583	6.00 ± 0.000	18.67	< 0.001	R vs RD: p < 0.001
						R vs C: Not Significant
						RD vs C: p < 0.001
8 hours	6.00 ± 0.000	5.63 ± 0.928	0.00 ± 0.000	1.3	0.276	No pairwise differences significant

Rescue Analgesia

In the present study, the mean time to rescue analgesia in group R was 300.71 ± 66.420 minutes; in group RD, it was 460.00 ± 141.903 minutes; and in group C, it was 137.14 ± 62.559 minutes. There was a statistically significant difference in the mean time to rescue analgesia between groups (p < 0.001) (Table 6).

**Table 6:** Time of requirement of first rescue analgesic Test used: One‑way ANOVA with Bonferroni post‑hoc correction was used for intergroup comparisons

Variable	Group R	Group RD	Group C	Test Statistic (F)	p‑value	Bonferroni Post‑Hoc Results
Time of Requirement of First Rescue Analgesic (minutes)	300.71 ± 66.420	460.00 ± 141.903	137.14 ± 62.559	52.83	< 0.001	R vs RD: p < 0.001
						R vs C: p < 0.001
						RD vs C: p < 0.001

## Discussion

Laparoscopic cholecystectomy is a minimally invasive procedure associated with moderate postoperative pain. The traditional approach to managing postoperative pain is multimodal, using non-steroidal anti-inflammatory drugs and opioids. Opioids are associated with nausea, vomiting, constipation, respiratory depression, urinary retention, and sedation [14]. A four-quadrant TAP block for postoperative analgesia helps reduce total opioid doses and, therefore, the side effects associated with opioids.

Studies assessing the TAP block for analgesia in laparoscopic cholecystectomy do not provide robust evidence to support its use for postoperative analgesia. Laparoscopic cholecystectomy is a day-care procedure. Postoperative pain management plays a major role in patient discharge decisions [15]. In this study, we administered four-quadrant TAP blocks. The four-quadrant TAP block reduces early postoperative pain following laparoscopic cholecystectomy by providing comprehensive analgesia to the entire abdominal wall. It covers the intercostal nerves (T6-T12) [16].

The hemodynamic parameters - intraoperative systolic blood pressure, diastolic blood pressure, heart rate, and mean arterial pressure - were more stable in patients in group R and RD than in group C. At one and four hours, VAS scores were significantly lower in group RD than in groups R and C (p < 0.001). At eight hours, there was no statistically significant difference among the groups (p = 0.276). There was no incidence of hypotension and bradycardia in any group.

The greater hemodynamic stability observed in Groups R and RD may be due to the analgesic and sympatholytic properties of the study drugs, which attenuated the surgical stress response and provided better perioperative pain control than in Group C. Intraoperative systolic blood pressure, diastolic blood pressure, mean arterial pressure, and heart rate remained comparatively stable in Groups R and RD.

In Group R and RD, ropivacaine provided effective analgesia by blocking the transmission of pain impulses through sensory nerve fibres, thereby maintaining cardiovascular stability throughout the intraoperative period.

The hemodynamic stability in Group RD may be further explained by the addition of dexmedetomidine. Dexmedetomidine is a highly selective α2-adrenergic agonist that produces sedation, analgesia, and sympatholysis. It suppresses catecholamine release in response to surgical stimulus [17]. Furthermore, dexmedetomidine potentiates the analgesic effect of local anaesthetics and reduces perioperative anaesthetic and opioid requirements. These combined effects likely contributed to the lower postoperative VAS scores observed at one and four hours in Group RD.

Although dexmedetomidine is known to potentially cause bradycardia and hypotension, such adverse effects were not encountered in this study, possibly due to the low dose administered and gradual drug absorption.

The analgesic benefits of the interventions gradually decreased over time as the drugs' pharmacological effects diminished, resulting in comparable pain perception (VAS score) among the three groups at eight hours.

A 2023 study by Çevikkalp et al. showed that a bilateral four-quadrant TAP block reduces early postoperative pain and opioid use after laparoscopic cholecystectomy [18]. They concluded that the four-quadrant TAP block technique is more effective than spinal anaesthesia, local anaesthetic infiltration, and bilateral two-quadrant TAP block in preventing early postoperative pain after laparoscopic cholecystectomy. A four-quadrant TAP block provides better pain control in the first 24 hours [18].

In a study by Suseela et al., ultrasound-guided bilateral subcostal transversus abdominals plane block and port-site infiltration were compared for postoperative pain management after laparoscopic cholecystectomy. They concluded that the subcostal TAP block provides superior analgesia [19].

A 2021 meta-analysis by Wang et al. assessing the efficacy of a TAP block for pain control in laparoscopic cholecystectomy concluded that the TAP block is superior to conventional pain management strategies in this procedure, but not superior to port-site infiltration. They recommended that more studies are needed to investigate the role of the TAP block in laparoscopic cholecystectomy [20].

Bansal et al., in their 2021 meta-analysis, reported that dexmedetomidine, when used as an adjuvant to ropivacaine in a TAP block after abdominal surgeries, enhanced the duration of postoperative analgesia and significantly delayed the time to first rescue analgesic request, thereby improving postoperative pain management [21].

A study conducted by Singla et al. in 2019 demonstrated that adding dexmedetomidine to local anaesthetics for TAP block following lower abdominal surgeries significantly reduced 24-hour opioid consumption and prolonged the time to first rescue analgesia, without increasing the incidence of adverse events [22].

The prospective randomised design, standardised anaesthetic protocol, and objective assessment of hemodynamic parameters are the major strengths of the study. Furthermore, the study evaluated clinically relevant outcomes, increasing its applicability in routine anaesthesia practice. There are certain limitations of the present study. This is a single-centre study with a small sample size, which limits its generalisability. The incidence of nausea and vomiting was not recorded during this trial. Pain assessment was performed using the VAS score, which is subjective and influenced by patients' perceptions and psychological factors. Furthermore, patient satisfaction was not assessed in this study.

## Conclusions

The present study demonstrates that a four-quadrant ultrasound-guided TAP block with ropivacaine and dexmedetomidine is an effective and safe technique for patients undergoing laparoscopic cholecystectomy. The addition of dexmedetomidine improved intraoperative hemodynamic stability and reduced postoperative pain scores. Therefore, dexmedetomidine may be considered a useful adjuvant to ropivacaine in TAP blocks as part of multimodal analgesia strategies for laparoscopic abdominal surgeries. However, the evidence is preliminary and requires confirmation in larger, multi-centric trials with extended follow-up and systematic safety monitoring.
